# Impact of *Phanerochaete chrysosporium* on the Functional Diversity of Bacterial Communities Associated with Decaying Wood

**DOI:** 10.1371/journal.pone.0147100

**Published:** 2016-01-29

**Authors:** Vincent Hervé, Elodie Ketter, Jean-Claude Pierrat, Eric Gelhaye, Pascale Frey-Klett

**Affiliations:** 1 INRA, Interactions Arbres–Microorganismes, UMR1136, F-54280 Champenoux, France; 2 Université de Lorraine, Interactions Arbres–Microorganismes, UMR1136, F-54500 Vandoeuvre-lès-Nancy, France; 3 INRA, UMR 1092 LERFOB, F-54280 Champenoux, France; 4 AgroParisTech, UMR 1092 LERFOB, F-54000 Nancy, France; USDA Forest Service, UNITED STATES

## Abstract

Bacteria and fungi naturally coexist in various environments including forest ecosystems. While the role of saprotrophic basidiomycetes in wood decomposition is well established, the influence of these fungi on the functional diversity of the wood-associated bacterial communities has received much less attention. Based on a microcosm experiment, we tested the hypothesis that both the presence of the white-rot fungus *Phanerochaete chrysosporium* and the wood, as a growth substrate, impacted the functional diversity of these bacterial communities. Microcosms containing sterile sawdust were inoculated with a microbial inoculum extracted from a forest soil, in presence or in absence of *P*. *chrysosporium* and subsequently, three enrichment steps were performed. First, bacterial strains were isolated from different microcosms previously analyzed by 16S rRNA gene-based pyrosequencing. Strains isolated from *P*. *chrysosporium* mycosphere showed less antagonism against this fungus compared to the strains isolated from the initial forest soil inoculum, suggesting a selection by the fungus of less inhibitory bacterial communities. Moreover, the presence of the fungus in wood resulted in a selection of cellulolytic and xylanolytic bacterial strains, highlighting the role of mycospheric bacteria in wood decomposition. Additionally, the proportion of siderophore-producing bacteria increased along the enrichment steps, suggesting an important role of bacteria in iron mobilization in decaying-wood. Finally, taxonomic identification of 311 bacterial isolates revealed, at the family level, strong similarities with the high-throughput sequencing data as well as with other studies in terms of taxonomic composition of the wood-associated bacterial community, highlighting that the isolated strains are representative of the wood-associated bacterial communities.

## Introduction

Microorganisms have been described as the main wood decomposers in forest ecosystems [1]. By using a plethora of extracellular lignocellulolytic enzymes [2–4], white-rot fungi are among the major actors of this wood decay [5–8]. While they are less studied, bacteria are also part of the decaying-wood microbial diversity [9] and are expected to contribute to wood decay. Indeed, tree species and wood decay class have been shown to significantly shape the structure of wood-inhabiting bacterial communities [10] and numerous bacteria present the capability of decomposing wood carbohydrates such as cellulose [11–12] and hemicelluloses [13–14]. Moreover, a few bacteria have also been described as being involved in lignin degradation [15–17].

In numerous environments, bacteria and fungi coexist and are known to interact physically and functionally [18–19], such as on decaying-wood in forests [20–21]. Aside from the environments, these interactions drive the establishment of specific bacterial communities in the surrounding areas of the fungal hyphae, called the mycosphere [22]. In the case of white-rot fungi, such a mycosphere effect on soil and wood-associated bacterial community composition has already been described. The presence of either *Resinicium bicolor*, *Hypholoma fasciculare* or *Phanerochaete chrysosporium* in both polluted and unpolluted soils has been shown to change the soil bacterial community composition [23]. The presence of *R*. *bicolor* and *H*. *fasciculare* has also been shown to reduce the number of bacteria colonizing wood blocks on forest soil and to modify the composition of these bacterial communities [24]. Concerning the white-rot fungus *P*. *chrysosporium*, the community composition of wood-associated bacteria has also been shown to be significantly modified by the presence of the fungus [25]. Wood, as a substrate, also shaped the taxonomic composition of wood-inhabiting bacterial communities [25]. Altogether these results raise the question of the functional diversity of the bacterial communities selected by the wood substrate and / or the white-rot fungus and more specifically, of their potential direct or indirect involvement in the wood decay process. In other environments, it was demonstrated that mycospheric bacterial communities had a functional potential significantly different from those of non-mycospheric bacterial communities. For example, in the mycosphere of ectomycorrhizal fungi, bacteria have been shown to present distinct functional diversity compared to the surrounding bulk soil [26–27]. Concerning the wood-decaying fungi, non-random species co-occurrence patterns among these fungi and nitrogen-fixing bacteria have been described in dead wood in temperate forests [28] but no deep analysis of the functional diversity of the bacterial communities has been performed. Furthermore, in the particular case of the white-rot mycosphere, whether a specific functional diversity of the bacterial communities is selected by the white-rot fungus during the wood decay process still remains an open question.

To address this question, we explored the functional diversity of the bacterial communities associated with decaying-wood, using a microcosm experiment with sawdust as growth matrix. A bacterial community (E0) was extracted from a forest soil and then inoculated in these microcosms, in presence (BF) or in absence (B) of the white-rot fungus *P*. *chrysosporium*. Three enrichment steps (E1, E2 and E3) were subsequently performed to create a favorable environment for the coexistence of the bacterial communities with wood and *P*. *chrysosporium*. It was previously demonstrated that specific bacterial communities were selected by decaying wood in presence of the white-rot fungus *P*. *chrysosporium* [25]. Thus, we isolated bacterial strains from various microcosms and investigated the functional diversity of these communities by a cultivation approach on different selective media. Indeed, such an approach is relevant to characterize potential functions in bacteria [29] and thus, understand their contribution to ecosystem functioning. The selective media were chosen in relation to the characteristics of the wood decay process. We isolated and identified 311 bacterial strains from the different treatments, *i*.*e*. presence or absence of *P*. *chrysosporium* at the different enrichment steps and initial soil inoculum. A randomly selected subset of 125 strains was then used for phylogenetic analysis and their functional potential was evaluated on selective media for siderophore production, ligninolytic, cellulolytic and xylanolytic activities. Their metabolic profile and their impact on the *in vitro* growth of *P*. *chrysosporium* were also characterized. All these assays were performed in order to advance understanding of the potential role of the bacterial communities from the white-rot mycosphere during the wood decay process. Lastly, the taxonomy of wood-associated bacteria is discussed.

## Materials and Methods

### Experimental design

Experimental design has been described in a previous study [25] (S1 Fig). Briefly, a microbial suspension was extracted from soil samples (cambisol) collected in a forest dominated by beech [30] using sterilized core borers (20 cm depth), after removing the overlying litter. Microorganisms were extracted using Nycodenz (Axis Shield, Oslo, Norway) density gradient (1.3 g.ml^-1^) centrifugation, according to Lindahl & Bakken [31]. The microbial suspension highly enriched in bacteria obtained during this step, called E0, was used as an initial microbial inoculum for the microcosm assay. The microcosm assay consisted of three successive enrichment steps in microcosms (140 mm Petri dishes sealed with adhesive tape) filled with 75 cm^3^ of beech (*Fagus sylvatica* L.) sawdust sieved (2 mm mesh) and autoclaved (20 min, 120°C) twice, with 2 days in between. Fungal inocula were prepared on beech wood blocks (50×30×10 mm). First, wood blocks were sterilized (autoclaved twice, 20 min, 120°C, with 2 days in between). The initial sterility of the sawdust and of the wood blocks was checked by plating woodblock suspensions at different dilutions on 10% TSA medium and by incubating them for 7 days at 25°C. No microbial growth was detected. The wood blocks were then incubated with *Phanerochaete chrysosporium* RP78 in Petri dishes, containing malt (30 g.l^-1^) agar (20 g.l^-1^) medium for 19 weeks, at 25°C, in dark conditions.

The microcosms were inoculated with two different microbial inocula, corresponding to two different treatments. In one treatment–called BF (for bacteria + fungus *ie*. *P*. *chrysosporium*)–the microbial suspension E0 (16 ml) was spread carefully over all of the sterile sawdust after having placed a beech block colonized by the white-rot fungus *P*. *chrysosporium* in the center of the microcosm. In a second treatment–called B (for bacteria, *ie* without *P*. *chrysosporium*)–the microbial suspension E0 (16 ml) was spread carefully over all of the sterile sawdust and a sterile beech wood block was placed over the mixture in the center of the microcosm. For the first step (E1), microcosms were incubated during 12 weeks to allow the establishment of fungal colony and / or bacterial communities. At the end of this period (E1), a 2 cm diameter round punch was used to sample 1.54 cm^3^ of the sawdust with associated bacteria and/or fungal hyphae. The sampling was conducted at four equally distant sites within each microcosm. The 4×1.54 cm^3^ of sawdust were then pooled and homogenized together. Half of it was used to inoculate sterile sawdust of a new microcosm and the other half was used to extract microbial suspensions by shaking vigorously for 1 min, 0.2 g of sawdust in 3 ml of sterile distilled water. All the microbial suspensions were plated on 10% TSA for bacteria enumeration after 48h at 25°C. All these microbial suspensions were then cryopreserved at -80°C in 25% glycerol prior to further analysis. Two further enrichment steps were performed every three weeks (E2 and E3 samples), using the same procedure for the three treatments. For B and BF treatments, ten replicates were made at each steps. All manipulations were done under sterile conditions, using a biosafety cabinet. All microcosms were sealed with adhesive tape and incubated at 25°C in the dark.

To monitor the functional diversity of both bacterial communities and strains over time, a subset of 21 microcosms was selected (the same ones that have been used in [25]): three for the B treatment for each step (E1, E2 and E3) and four for the BF treatment for each step. The bacterial diversity from the E0 inoculum was also analyzed. The presence of *P*. *chrysosporium* was checked for each BF microcosm using nested PCR as previously described [25]. Additionally, the presence of other fungi, originating from the E0 inoculum, was detected in all the samples by observations with light transmission microscopy and by plating microbial suspension on malt agar medium (see [25] for details)

### Collection of bacterial strains

A total of 311 bacterial strains were isolated from the 21 selected microcosms and the E0 inoculum as follow. One microbial suspension was prepared for each sample by shaking vigorously for 1 min, 0.2 g of sawdust in 3 ml of sterile distilled water. Different dilutions (from 10^−1^ to 10^−4^) of the suspensions were plated in duplicate on water yeast agar (WYA) [24] containing 50 mg.l^-1^ thiabendazole (Sigma) and 100 mg.l^-1^ cycloheximide (Sigma). WYA contained 1 g.l^-1^ NaCl, 0.1 g.l^-1^ yeast extract (Difco), 1.95 g.l^-1^ MES (Sigma), 20 g.l^-1^ agar and its pH was adjusted at pH 5. All plates were incubated at 25°C for 7 days in dark condition. To collect bacterial isolates showing a similar level of dominance within each sample, bacterial isolation from each sample was always performed with samples diluted to the same level as recommended by Frey et al [32]. Bacterial colonies were randomly selected to obtain about fourteen isolates per sample, transferred on WYA for isolation and then subcultured twice on 1/10-strength tryptic soy agar (TSA) medium (3 g.l^-1^ Tryptic Soy Broth from Difco and 15 g.l^-1^ agar). All the bacterial isolates were cryopreserved at -80°C in 25% glycerol. For each condition (*i*.*e*., enrichment step x fungal presence), 40 to 49 bacteria were isolated.

For all the functional assays on the bacterial strains described below, each strain was grown on 10% TSA medium for 24h at 25°C. Subsequently, the bacteria were collected in sterile distilled water and washed once before adjusting the absorbance at 595 nm of the suspension to 0.4, in order to obtain the bacterial inoculum.

### Identification of bacterial strains and phylogenetic analysis

An amplified fragment of the 16S rRNA gene with the universal set of primers pA (5′-AGAGTTTGATCCTGGCTCAG-3′) [33] and 907r (5′-CCGTCAATTCMTTTGAGTTT-3′) [34] was used to identify bacterial isolates. Boiled bacterial cells (10 min at 98°C) were directly used as a template in PCR reactions. PCR reactions were performed in a 50 μl final volume containing 20 μl Master Mix (5 PRIME, Germany), 24 μl water Mol Bio grade (5 PRIME, Germany), 2.5 μl of each primer (10 μM) and 1 μl DNA. The PCR conditions used were 94°C for 4 min, 30 cycles of 30 s at 94°C (denaturation), 53°C for 90 s (annealing) and 72°C for 90 s (extension), followed by 10 min at 72°C. PCR products were purified using MultiScreen HTS filter plates (Millipore, Ireland) and then sequenced using Sanger method at Eurofins MWG (Ebersberg, Germany). All sequences were identified using the EzTaxon-e server [35] on the basis of 16S rRNA gene sequence data. Accession numbers of partial 16S rRNA gene sequences have been deposited in GenBank under accession numbers KM604797 to KM605107.

All sequences were aligned using ClustalW version 2.1 [36]. A maximum likelihood phylogenetic tree was constructed using RAxML version 7.7.2 [37] with the GTRGAMMA model of DNA evolution. To obtain the statistical confidence of internal branches, 10000 bootstrapped trees were generated using RAxML. From this tree, unweighted UniFrac distances were computed [38] and unweighted pair group method with arithmetic mean (UPGMA) clustering was used to generate a dendrogram.

### Bacterial-fungal confrontations

A confrontation assay was performed in 90 mm diameter Petri dishes to evaluate bacterial-fungal interactions. The fungus *P*. *chrysosporium* RP78 was grown on malt (30 g.l^-1^) agar (20 g.l^-1^) medium at 25°C. A 6-mm diameter fungal plug of *P*. *chrysosporium* was taken from the margin of 1-week-old fungal colony and placed in the center of a WYA plate. Two 10-μl droplets of a bacterial suspension with A_595nm_≈0.4 were spotted at 3 cm from the fungal plug, diametrically opposed. Petri dishes were sealed with adhesive tape and incubated at 25°C, in dark condition. The diameters of the fungal colony in the direction of the bacterial colonies (D1) and the one orthogonal to D1 (called D2) were measured twice per day *i*.*e* 18, 28, 42, 52 and 66 hours after the inoculation, until the fungal colonies reached the periphery of the plates. Then the ratio R = D1/D2 was used to estimate the influence of each bacterial strain on *P*. *chrysosporium* growth. For each confrontation, *n* = 5 replicates were made. As a control, fungal growth in absence of bacteria was also measured in the same condition.

### Selective media and metabolic assays

To characterize the functional potential of the bacterial strains, 125 strains from the 311 strain collection were randomly selected from five different treatments: E0, E1B, E1BF, E3B and E3BF (*n* = 25 per treatment) (S1 Table). Five selective media were used: (*i*) the WYA medium, (*ii*) a WYA medium containing 0.05% Remazol Brilliant Blue R (Sigma), (*iii*) a medium containing carboxymethyl-cellulose (CMC) as sole source of carbon (K_2_HPO_4_ 1.0 g.l^-1^; (NH_4_)_2_SO_4_ 1.0 g.l^-1^; MgSO_4_,7H_2_O 0.5 g.l^-1^; NaCl 0.5 g.l^-1^; carboxymethyl-cellulose sodium salt (Sigma) 5 g.l^-1^; agar 20 g.l^-1^; pH 5) [39], (*iv*) a medium containing xylan as sole source of carbon (K_2_HPO_4_ 1.0 g.l^-1^; (NH_4_)_2_SO_4_ 1.0 g.l^-1^; MgSO_4_,7H_2_O 0.5 g.l^-1^; NaCl 0.5 g.l^-1^; beechwood xylan (Sigma) 10 g.l^-1^; agar 20 g.l^-1^; pH 5) and (*v*) a chrome azurol S (CAS) agar medium prepared following the method of Alexander et al. [40]. For each bacterial strain to be tested, 10-μl droplets of a bacterial suspension with A_595nm_≈0.4 were spotted in triplicate in one Petri dish of each medium. Petri dishes were incubated 7 days at 25°C in dark condition. Lignolysis was indicated by the Remazol Brilliant Blue R (RBBR) medium turning from blue to pale pink [41]. The cellulolytic activity on CMC medium and xylanolytic activity on xylan medium were detected using 0.1% Congo red (Sigma) for staining during 40 min followed by a washing with 1M NaCl according to the Teather et al. method [42]. For the siderophore production assay on CAS medium, the discoloration of the medium (blue to yellow or orange) indicated siderophore-producing bacterial strains.

Metabolic fingerprint of the same 125 bacterial strains was performed using Biolog GN2 microplate according to the manufacturer's instructions. Microplates were inoculated with 150 μl of a bacterial suspension with A_595nm_≈0.4 and incubated 24h at 25°C in dark condition. Then, color density of each well was measured at 595 nm using a iMark Microplate Absorbance Reader (Bio-Rad, Hercules, CA, USA). Data were normalized by dividing the raw difference value for each well by the average well color development (AWCD) of the plate, as suggested by Garland et al. [43]. Negative values were considered as 0 in subsequent data analyses. Shannon index was used to compute the diversity of carbon substrate utilization for each strain [44].

### Statistical analyses

The ability of the 125 bacterial strains to grow on selective media and to use them, was analyzed using generalized linear models (glm) under a binomial distribution. Differences in the utilization of the 95 carbon substrates of the Biolog GN2 microplate among treatments (enrichment step x presence of the fungus) were evaluated by a Permutational Multivariate Analysis of Variance (PERMANOVA), using the *adonis* function from the *vegan* package in R with a Bray-Curtis dissimilarity matrix and 100000 permutations. Subsequently, the utilization of each substrate was estimated by a one-way ANOVA followed by the Tukey HSD *post hoc* test. All statistical analyses and graphics were computed using R software version 3.0.3 [45].

### Comparison of different 16S rRNA gene data sets

In order to compare results from cultivation and pyrosequencing methods, an operational taxonomic unit (OTU) based approach was performed using the Mothur software version 1.29.1 [46]. 16S rRNA gene pyrosequencing reads (accession numbers SRR900229 to SRR900250 in GenBank) were processed by largely following the Schloss standard operating procedure [47], as previously described [25]. Briefly, sequencing errors were reduced by implementation of the AmpliconNoise algorithm and low-quality sequences were removed (minimum length 200 bp, allowing 1 mismatch to the barcode, 2 mismatches to the primer, and homopolymers no longer than 8 bp). Sequences were then trimmed to keep only high quality reads (Q ≥ 35). The 16S rRNA gene sequences of the 311 bacterial strains were also handled with Mothur. The sequences of these two data sets were aligned in Mothur and classified against the SILVA bacterial SSU reference database v102 [48]. Chimera were removed using the chimera.uchime mothur command. Singletons were included in the analysis. Finally, all the sequences were assigned to family-level phylotypes using the naïve Bayesian classifier implemented in Mothur and clustered into OTUs using the average neighbor method. An OTU was defined at the 97% sequence similarity level.

In order to compare our 16S rRNA gene sequences from the cultivated bacteria with other sequences from studies related to bacterial communities associated with decaying wood [21,24,49,50], the RDP Library Compare tool version 2.6 with 95% confidence threshold was used [51]. The E0 sample, corresponding to the bacteria extracted from a forest soil, was excluded from this analysis.

## Results and Discussion

### Phylogenetic structure of the isolated bacterial strains

**Fig 1:**
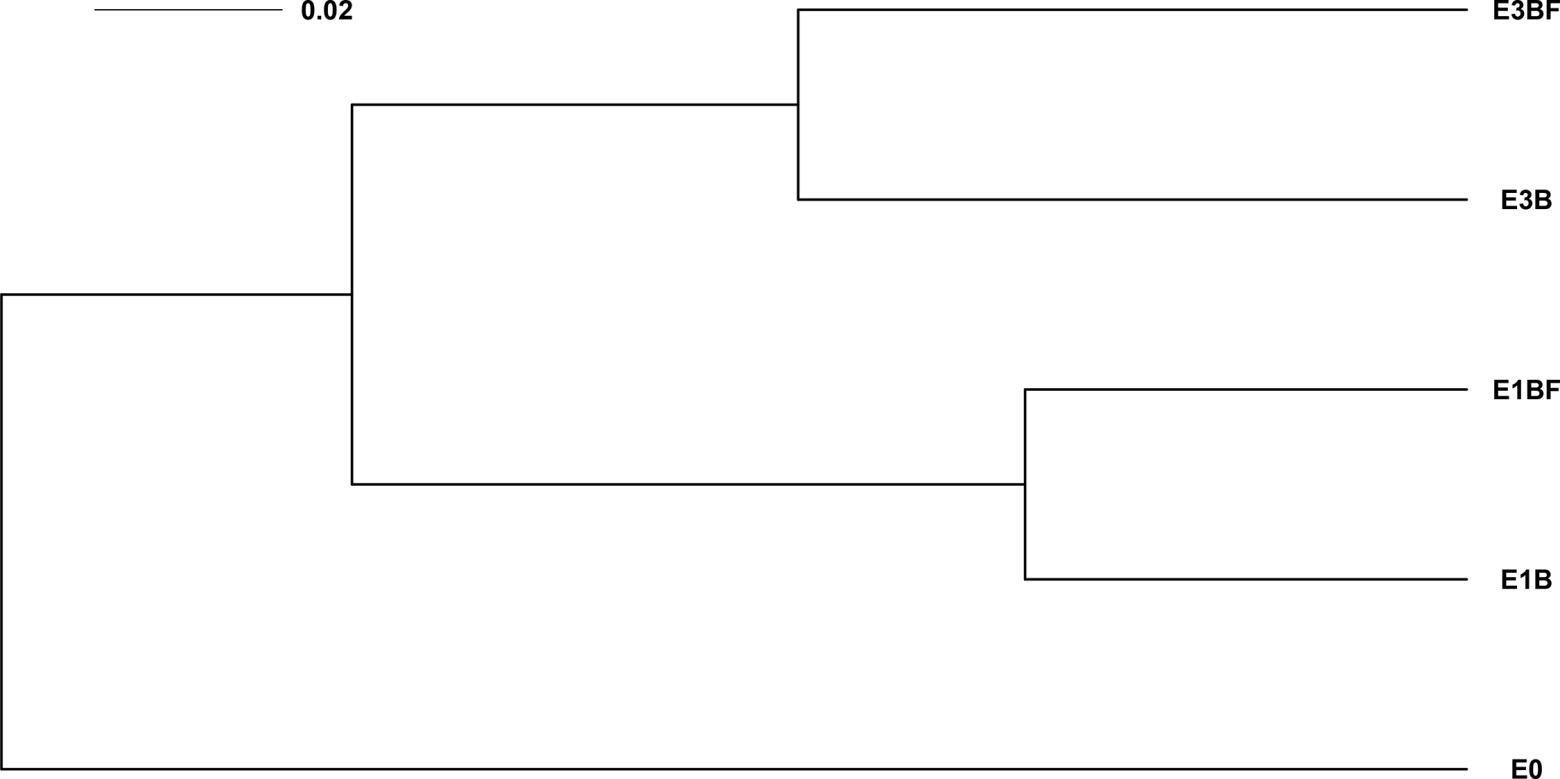
16S rRNA gene-based dendrogram generated from the UPGMA clustering based on unweighted UniFrac distances. Branches are proportional to this distance. *n* = 25 sequences per treatment.

A total of 311 bacterial strains were isolated from the different treatments and identified by sequencing a fragment of the 16S rRNA gene (S1 Table). Among these strains, we randomly selected 125 strains sampled in five different treatments (*n* = 25) for further functional analyses. Because we wanted to focus on both wood and mycosphere effects, we chose to work with strains from E0, E1B, E1BF, E3B and E3BF treatments only (S1 Table). Based on a fragment of the 16S rRNA gene, we built a maximum likelihood phylogenetic tree, to then compare the phylogenetic distances between these five different treatments. UPGMA clustering based on unweighted UniFrac distance revealed a clear distinction between the initial inoculum E0 and the samples from wood-based microcosms, a separation between the enrichment steps (E1 and E3) and finally a separation between the samples inoculated with or without *P*. *chrysosporium* (BF and B respectively) (Fig 1). A similar clustering was observed with a 16S rRNA gene-based pyrosequencing approach performed on the same samples [25]. This highlights that in the case of wood-associated bacteria, culture-dependent and culture-independent approaches can result in a similar phylogenetic pattern for the bacterial communities. Interestingly, such results were also observed for forest soil bacterial communities [52]. Since at least a part of wood-associated bacteria is culturable, it gives the opportunity of studying the functional diversity of this community by analyzing the functional potential of some of the bacterial strains that reflect the overall phylogenetic structure of the community. Taxonomic assignment of these strains will be discussed below.

### Bacterial-fungal confrontations

**Fig 2:**
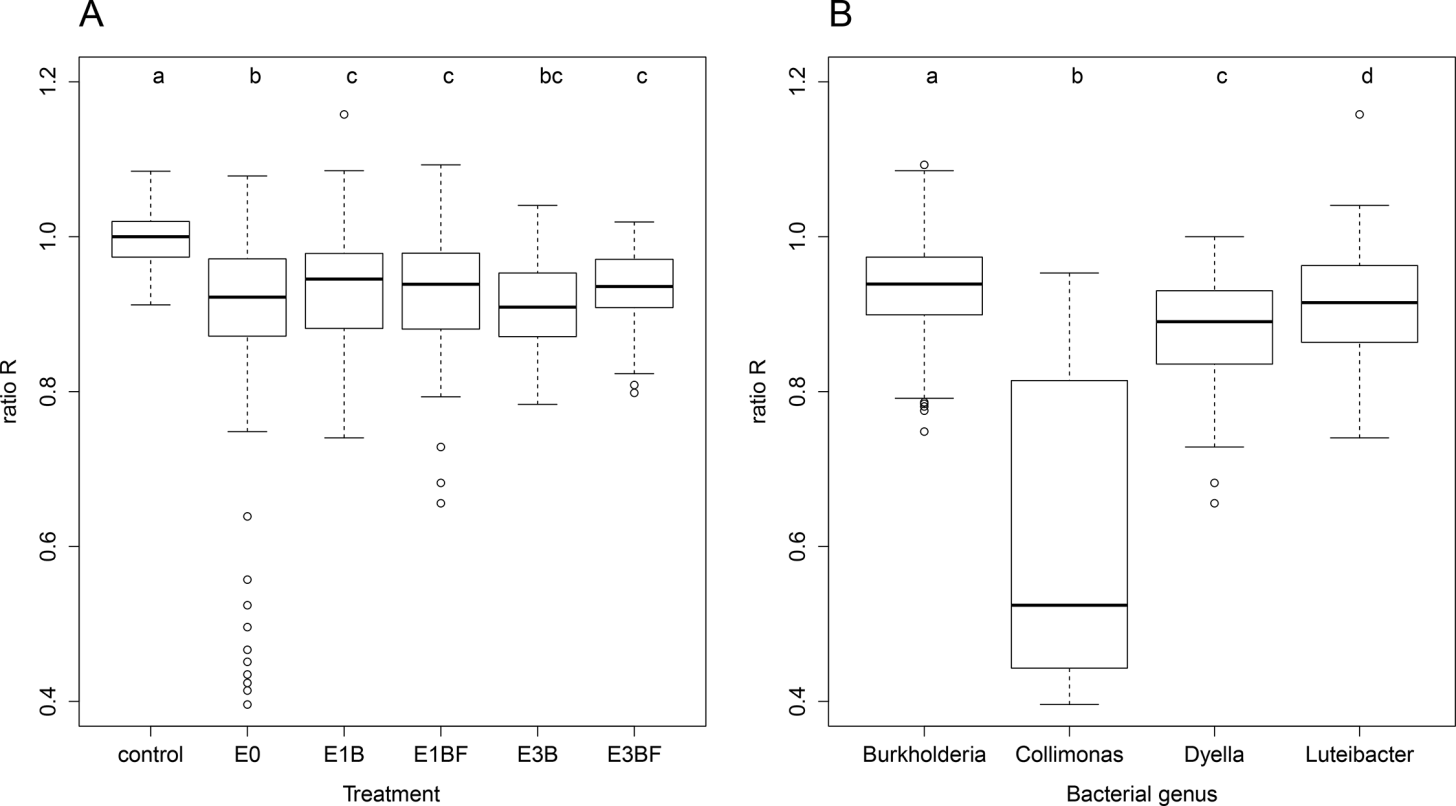
Bacterial-fungal confrontations after 66 hours of incubation. Different letters above boxplots indicate significant difference based on ANOVA followed by Tukey's HSD test (*p*<0.05). **A.** Ratio R (ratio of the diameter of the fungal colony growing in the direction of the bacterial colonies over the diameter of the colony growing in the orthogonal direction, see Materials and Methods) of the fungal growth in presence of one bacterial strain, as a function of the treatment. *n* = 25 bacterial strains per treatment with 5 replicates per strain. Control corresponded to *P*. *chrysosporium* growing alone. **B.** Relationships between the genus of the bacterial strains and the effect on the fungal growth based on the R ratio. For *Burkholderia*, *n* = 90 strains; for *Collimonas*, *n* = 3; for *Dyella*, *n* = 8; for *Luteibacter*, *n* = 23. *Cupriavidus* was excluded from the analysis because *n* = 1.

Confrontation assays between the 125 selected bacterial strains and *P*. *chrysosporium* were performed to evaluate the effect of these strains on the fungal growth. The fungal growth, here estimated by the R ratio (ratio of the diameter of the fungal colony growing in the direction of the bacterial colonies over the diameter of the colony growing in the orthogonal direction, see Materials and Methods section), was significantly affected by the treatments (enrichment step x fungal presence) (one-way ANOVA, F_5, 649_ = 10.37, *p*<0.001) and by the genus of the bacterial strains (one-way ANOVA, F_3, 616_ = 119.20, *p*<0.001) (Fig 2). Compared to *P*. *chrysosporium* growth in axenic condition, *P*. *chrysosporium* growth in presence of bacterial strains from the different treatments (enrichment step x fungal presence) was significantly lower (*p*<0.002), indicating antagonism between the two types of microorganisms in this *in vitro* assay (Fig 2A). However, fungal growth inhibition was significantly less pronounced (*p*<0.05) with the bacterial strains isolated in *P*. *chrysosporium* mycosphere, *i*.*e*. isolated from the E1BF and E3BF samples, compared to the bacterial strains isolated from the forest soil inoculum E0, suggesting a selection by the fungus of less inhibitory bacterial communities. Similarly, in the case of the Douglar fir-*Laccaria bicolor* ectomycorrhizal symbiosis, it has been reported that the proportion of *Pseudomonas fluorescens* strains inhibiting this symbiosis was significantly lower in the mycosphere of *L*. *bicolor* compared to the strains isolated from the bulk soil of a forest nursery [26]. Interestingly, no antagonism was detected between the white-rot fungus *Hypholoma fasciculare* and bacterial strains isolated from its mycosphere, as revealed by *in vitro* confrontation assays [21]. These assays were however performed on a nutrient-rich 1/10 TSA medium where nutritive competition is less likely to occur, whereas our assays were performed on the minimal medium WYA. The differences in the results obtained could be attributed to the growth medium composition, which is known to affect fungal growth during confrontations with bacteria [53]. Our results also revealed a relationship between the fungal growth and the genus of the bacterial strains used in the confrontations (Fig 2B). Strains belonging to the *Collimonas* genus reduced the most the fungal growth and were only found in the forest soil inoculum E0 (S1 Table). This inhibition of fungal growth or fungistasis has already been described as a characteristic of this genus [54]. Interestingly, no member of this genus was isolated or detected by 16S rRNA gene-based pyrosequencing in the woody microcosms [25], suggesting that *Collimonas* might not be adapted to wood environment. This result is in accordance with the oligotrophic lifestyle of this bacterial genus usually found in lower carbon content environments compared to decaying-wood, such as dune, forest or tundra soils [54]. On the contrary, strains belonging to the *Burkholderia* genus had the lowest impact on *P*. *chrysosporium* growth and were isolated in all samples, suggesting tolerance of this bacterial genus to the fungus. Interestingly, this tolerance to the presence of fungi was previously mentioned in a study of the biogeography of *Burkholderia* populations [55].

### Functional potential of the bacterial strains

**Fig 3:**
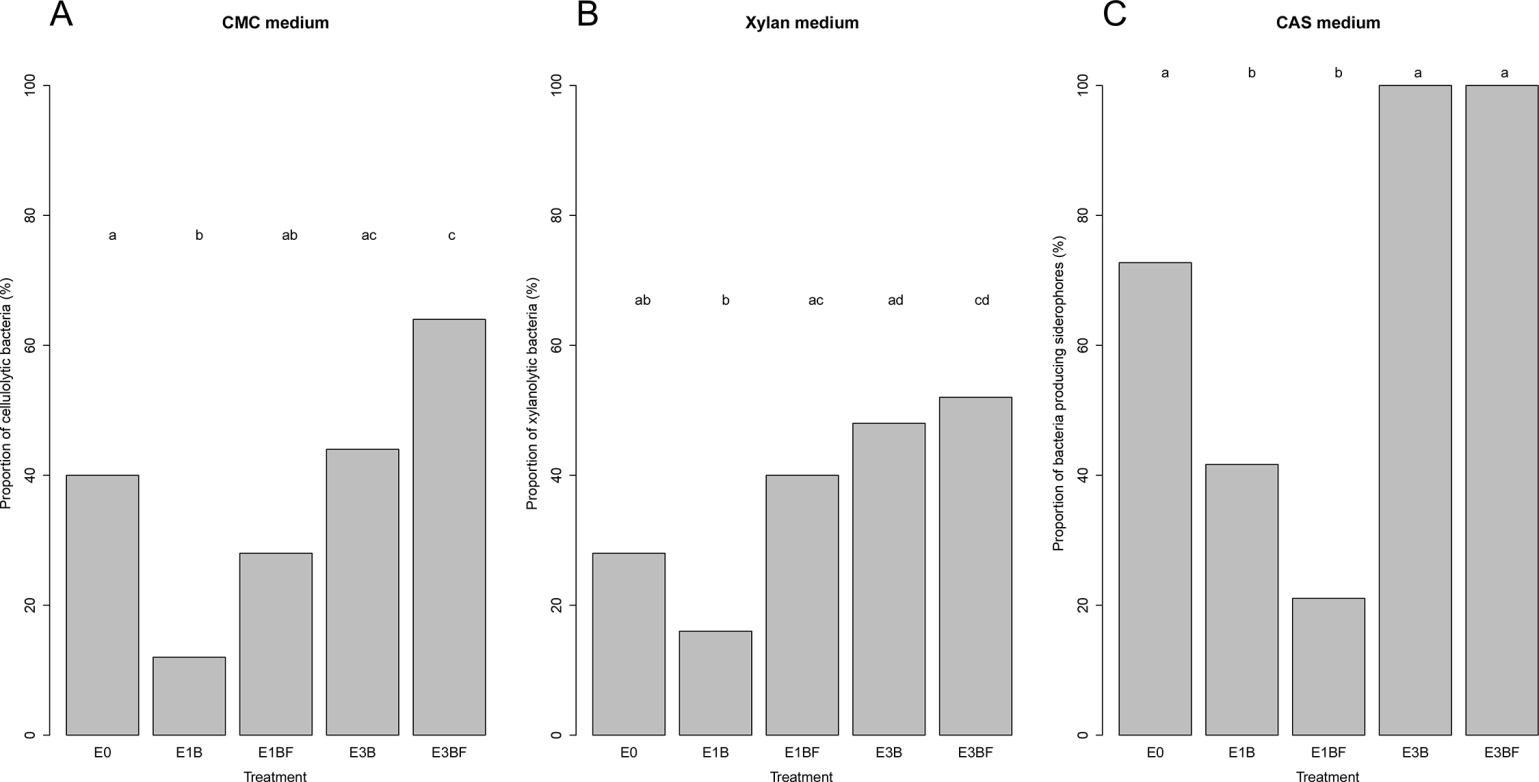
Capabilities of the strains able to grow on the selective media to use different substrates. Different letters above barplots indicate significant difference based on glm under a binomial distribution (*p*<0.05). **A.** Cellulolytic activity on carboxymethyl-cellulose medium. *n* = 25 bacterial strains per treatment with 3 replicates per strain. **B.** Xylanolytic activity on beechwood xylan medium. *n* = 25 bacterial strains per treatment with 3 replicates per strain. **C.** Siderophore production assay on CAS medium. *n* = 22 bacterial strains for E0, *n* = 10 for E1B, *n* = 18 for E1BF, *n* = 19 for E3B, *n* = 22 for E3BF; with 3 replicates for each strain. Variation in the number of strains is due to the fact that not all strains could grow on CAS medium.

To address the question of the functional role of the bacterial strains in the process of wood degradation, notably regarding the degradation of three main wood components, namely cellulose, hemicellulose and lignin, the functional potential of 125 bacterial strains was studied using different selective media. Because iron plays a role in the oxidative process of wood degradation and wood-decaying *Basidiomycetes* are known to produce siderophores [56] potentially involved in lignocellulose degradation [57], the production of siderophores was also evaluated for the 125 selected bacterial strains. All screened strains were able to grow on WYA (control), Remazol Brilliant Blue R (RBBR), carboxymethyl-cellulose (CMC) and xylan media, regardless of the treatment in which they were sampled. Concerning the ligninolytic activity, none of the bacterial strains was able to degrade RBBR, whereas *P*. *chrysosporium* was (positive control). Similar results were found for bacteria isolated on spruce stumps in a previous study [41], suggesting an absence of ligninolytic activity for the bacterial isolates alone. Concerning the cellulolytic potential of the bacterial strains, there was no effect of *P*. *chrysosporium* on the proportion of bacterial strains able to degrade CMC, but there was an enrichment effect leading to a significant increase (glm, *p*<0.05) in cellulolytic bacteria proportion from the first (E1) to the third (E3) step of the enrichment (Fig 3A). Regarding the number of xylanolytic bacterial strains, the effect of *P*. *chrysosporium* was only noticeable at the first step of the enrichment with a higher proportion of xylanolytic bacteria (glm, *p*<0.02) in presence of the fungus (E1BF) (Fig 3B), indicating a mycosphere effect of *P*. *chrysosporium* after twelve weeks of incubation with the selection of hemicellulose-degrading bacteria. There was also a significant increase (glm, *p*<0.05) in xylanolytic bacteria proportion from the first (E1) to the third (E3) step of the enrichment. This shows that an enrichment procedure in a woody environment allows isolating efficient cellulose and hemicellulose-degrading bacterial strains. More precisely, compared to the initial inoculum E0, the number of cellulolytic and xylanolytic bacterial strains isolated from E3BF samples was significantly higher (glm, *p*<0.05), showing that the combined effects of the enrichment procedure in wood and the presence of a white-rot fungus resulted in a selection of cellulose and hemicellulose-degrading bacterial strains that are expected to contribute to the wood decay process. Such results raise the question of a potential synergy between a white-rot fungus and some mycospheric bacterial strains in the process of wood decomposition. In the case of *Pinus sylvestris* decomposition by *Hypholoma fasciculare* in contrast, it has been reported that the presence of mycospheric bacterial isolates did not influence the wood decomposition rate [58]. Concerning the siderophore production assay on CAS medium, not all the bacteria were able to grow on this medium (22 strains able to grow on 25 tested; 10/25; 18/25; 19/25 and 22/25 for E0, E1B, E1BF, E3B and E3BF, respectively). There was no effect of *P*. *chrysosporium* on the proportion of bacterial strains able to produce siderophores, but there was an enrichment effect with a significant increase (glm, *p*<0.05) in siderophore-producing bacteria proportion from the first (E1) to the third (E3) step of the enrichment, leading to the fact that all strains from E3 able to grow on CAS medium were able to produce siderophores (Fig 3C). This clearly suggests that bacteria might play an important role in iron mobilization in woody environment, regardless of the presence of *P*. *chrysosporium*. Interestingly, the production of siderophores by bacterial isolates from decaying *Picea sitchensis* has also been reported [41].

**Fig 4:**
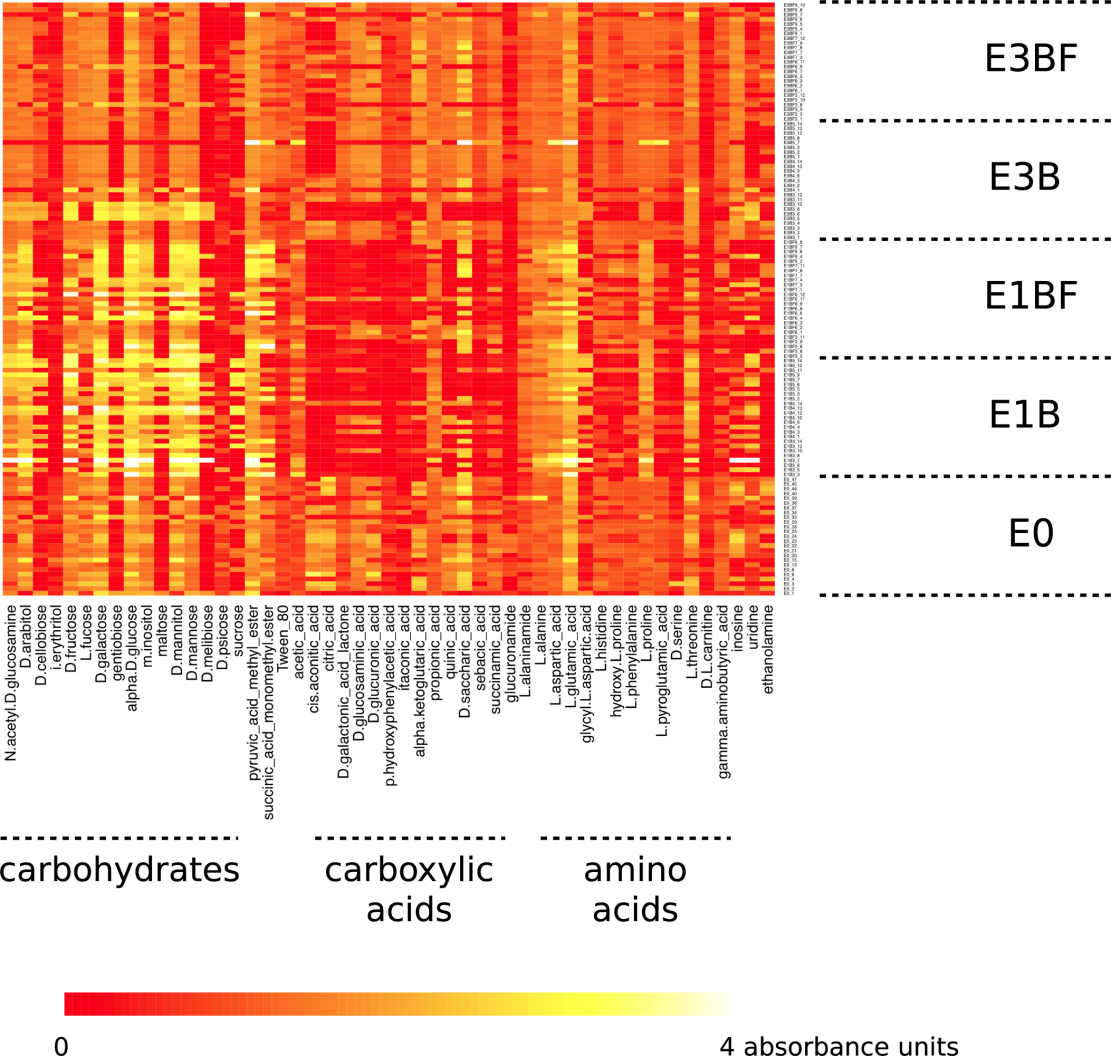
Metabolic profiles of 125 bacterial strains randomly selected in the 5 treatments (E0, E1B, E1BF, E3B, E3BF) (*n* = 25). The heatmap shows the 51/95 substrates with statistically significant differences between treatments (enrichment step E x presence (BF) or absence (B) of *P*. *chrysosporium*) (ANOVA, *p*<0.05).

Finally, to better characterize the functional potential of these 125 bacterial strains and thus to clarify their roles, metabolic profiles were performed using Biolog GN2 microplates. Each microplate contains 95 different carbon substrates, including some carbon compounds produced by the microbial degradation of wood and compounds synthesized by some fungi. Globally, the utilization of these 95 carbon substrates was significantly affected by the treatment (enrichment step x presence of the fungus) (PERMANOVA, df = 4, F = 7.20, *p*<0.001), indicating differences of the functional potential of the bacterial strains isolated from the five different niches, *i*.*e*. E0, E1B, E1BF, E3B or E3BF. Diversity of carbon substrate utilization, based on Shannon index [44], increased significantly from E1 to E3 (Kruskal-Wallis test, *p*<0.01), regardless of the presence of the fungus (S2 Fig), showing the enrichment effect in a woody environment on the functional diversity of these bacterial strains. Interestingly, the functional diversity of the strains isolated at the end of the enrichment (E3) was comparable with the functional diversity of the strains isolated from the forest soil (E0), revealing a physiological adaptation of the bacterial communities to a nutrient-poor environment, *i*.*e*. the wet sawdust. On the 95 carbon substrates, 51 were differentially used (one-way ANOVA, df = 4, *p*<0.05) according to the ecological niche where the strains were isolated, *i*.*e*. E0, E1B, E1BF, E3B or E3BF. The utilization of these 51 substrates mainly varied between the enrichment steps (Fig 4). But strains from E0, E3B and E3BF showed similar metabolic profiles. E1B and E1BF strains differed from them in the utilization of carbohydrates and carboxylic acids. Indeed, at the first step of the enrichment E1, carbohydrate utilization was higher, particularly for the D-fructose, D-galactose, alpha D-glucose, D-mannitol and D-mannose, while carboxylic acid utilization was lower, especially for the *cis*-aconitic acid and citric acid, compared to E0 and E3 treatments. Such an increase in the carbohydrate utilization in E1 might be explained by the fact that incubation time was longer (12 weeks) in E1 than in E3 (3 weeks), leading to a more advanced stage of wood decomposition and thus resulting in more monosaccharides available in the environment, since D-glucose is a product of cellulose degradation [59] and hemicellulose contains glucose, galactose and mannose. Altogether, these different results indicate that in a woody environment, bacteria have the potential for being active decomposers of both wood-carbohydrate polymers and monomers. Interestingly, for the first step of the enrichment E1, significant differences in the utilization of certain carbon substrates were observed between the bacterial strains isolated in presence (BF) and in absence of *P*. *chrysosporium* but none of these differences could be detected in the third step of the enrichment (E3B versus E3BF), confirming the strong mycosphere effect of *P*. *chrysosporium* on the bacterial strains after twelve weeks of incubation (E1) in woody microcosms. Among the carbohydrates, the utilization of D-cellobiose, D-fructose, gentiobiose, maltose and sucrose was significantly higher for the E1B strains compared to the E1BF strains (one-way ANOVA, *p*<0.05). These results might suggest, especially in the case of the cellobiose which is a product of cellulose degradation, a selection by the fungus of bacterial strains less efficient in wood-carbohydrate degradation.

### Taxonomy of the wood-associated bacteria

**Fig 5:**
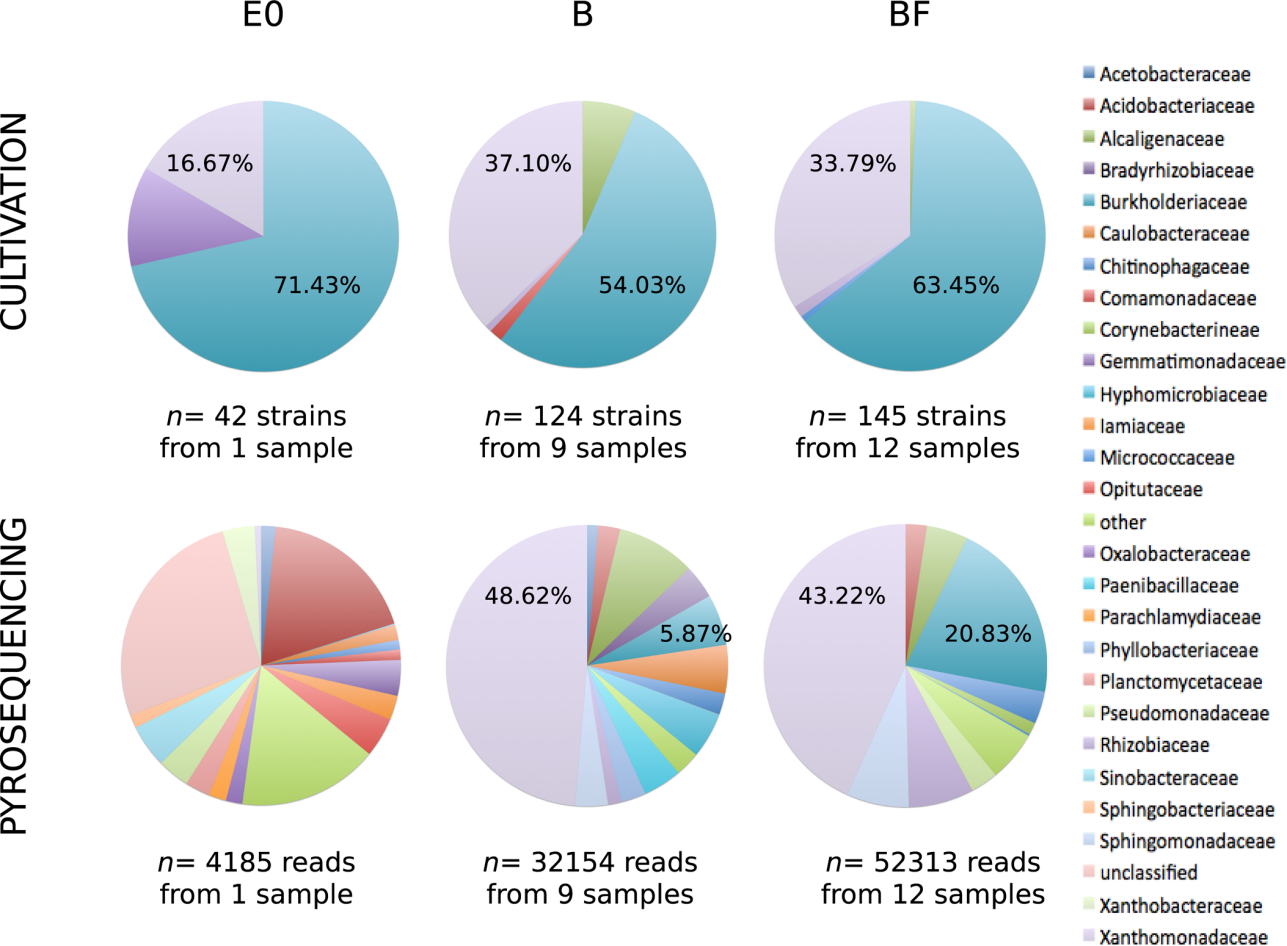
Pie charts comparing the taxonomic diversity of the bacterial OTUs from E0, B and BF samples at the family level, using culture-dependent and culture-independent (pyrosequencing) approaches [25]. Families that accounted for less than 1% in a sample are summarized in a category termed “other”.

In a previous study, the bacterial taxonomic diversity of the same 22 samples was explored using 16S rRNA gene-based pyrosequencing [25]. To evaluate the representativeness of the strains isolated in the present study, taxonomic assignments of the 16S rRNA sequences generated by both culture-dependent and culture-independent approaches were compared. Sequences were processed similarly and clustered into operational taxonomic units (OTUs, at 97% sequence similarity level). Sequences from the bacterial isolates (S1 Table) clustered into 14 OTUs while 3553 OTUs were obtained by 454 pyrosequencing. Subsequently three sample groups were considered: the initial inoculum E0 and the woody microcosms in absence (B) or in presence (BF) of *P*. *chrysosporium*, without taking into account the enrichment steps (Fig 5, S1 Fig). For the initial inoculum E0, cultivation and pyrosequencing approaches led to very different taxonomic patterns. This can be explained by the relatively low number of isolated bacterial strains (42 strains), but also by the fact that, even using pyrosequencing, it was not possible to describe the whole diversity in E0, as shown by the unsaturated rarefaction curve. On the contrary, from the woody environment in the microcosms (B and BF groups of samples) it was possible to isolate OTUs belonging to the two most abundant bacterial families detected by pyrosequencing, namely *Xanthomonadaceae* and *Burkholderiaceae*. Indeed, the OTUs belonging to the *Xanthomonadaceae* and *Burkholderiaceae* families represented together more than 54% and 64% of the relative abundance for B and BF treatments, respectively. Interestingly, OTUs of these two families were also the most abundant among the bacterial strain collection, representing together more than 91% and 97% of the isolated strains for B and BF treatments, respectively. OTUs belonging to *Burkholderiaceae* were found to be more abundant in BF samples than in B samples with the cultivation approach, as already observed with the pyrosequencing approach. Altogether, these results show that it is possible to isolate and cultivate the dominant members of the bacterial communities associated with decaying-wood. Vaninsberghe et al. [52] recently showed similar results in a study on forest soil bacterial communities in which they were able to isolate 22% of the OTUs detected by a pyrosequencing approach. Isolation of major representative bacterial strain from a particular ecological niche is very important since it allows to study the physiology and the functional role of the niche members and thus, to better understand the functioning of this niche.

To evaluate if the bacterial strains isolated in this study were phylogenetically related to already known wood-associated bacteria, we compared the taxonomic diversity of the present bacterial collection with the ones revealed in four other publications dedicated to bacterial communities associated to decaying wood, using either a culture-dependent approach [21,24,50] or a culture-independent one [21,49] (S2 Table). Because we wanted to compare data sets with similar number of sequences, we deliberately did not include studies based on high-throughput sequencing. This comparative analysis revealed that regardless of the studies and of the approaches, *Proteobacteria* was the major phylum of the wood-associated bacterial communities. This result is corroborated by a recent 16S rRNA gene-based pyrosequencing study in which the bacterial OTUs belonging to *Proteobacteria* were found to be dominant in wood cubes inoculated in a boreal forest [60]. More precisely, among *Proteobacteria* we found that members of the *Xanthomonadaceae* family were always present in these studies (S2 Table), indicating that *Xanthomonadaceae* are strongly associated with decaying-wood habitat. Interestingly, these different studies embrace both field [21,49] and microcosm experiments [24,50], as well as decaying wood of both conifers [21,49,50] and broadleaved trees [21,24,50], suggesting no host-tree specificity for *Xanthomonadaceae* colonizing wood. Overall, this comparison indicates that in the present work, we were able to isolate members of the wood-associated bacterial community, that we then functionally described.

## Conclusions

Using a microcosm-scale experiment, we observed that the functional diversity of the culturable wood-associated bacterial communities was affected by both *P*. *chrysosporium* and the wood substrate during the wood decay process. Moreover the functional characterization of the isolated bacterial strains highlighted that strains isolated from the mycosphere of *P*. *chrysosporium* were potentially involved in wood degradation, mainly via cellulolytic and xylanolytic processes. In terms of taxonomy, this study also revealed the ability to culture and isolate strains corresponding to representative members of the wood-associated bacterial communities.

## Supporting Information

S1 Fig Experimental design of the microcosm experiment. The diagram was adapted from [25]. E0, a microbial suspension extracted from forest soil, was used as the initial inoculum. E0 was mixed with sterile sawdust as growth matrix, in two conditions: including (BF) or not (B) the white-rot fungus *P*. *chrysosporium* previously inoculated on a beech wood block (F). After twelve weeks of incubation (E1), an enrichment was performed every three weeks (from E1 to E3), using a fraction of colonized sawdust sampled from a microcosm to inoculate a new sterile one.
(PDF)

S2 Fig Diversity of carbon substrate utilization for each treatment (*n* = 25) based on Shannon index. Different letters above boxplots indicate significant difference based on non-parametric Kruskal-Wallis test (*p*<0.05).
(PDF)

S1 Table Taxonomic assignment of the 311 strains isolated during the study. The assignment was performed using the EzTaxon-e server [35] and on the basis of 16S rRNA gene sequences (>800 nucleotides). The 125 strains randomly selected for functional characterization are highlighted in gray.
(PDF)

S2 Table Comparison of the taxonomic composition (in %) of bacterial communities associated with decaying wood from different studies. The comparison was based on 16S rRNA gene sequences and using the RDP Library Compare tool version 2.6 with 95% confidence threshold, except for Blomqvist et al. [50] in which no sequences were available but only taxonomic identification.
(PDF)
