# Supplementary figures and images for: Impact of Phanerochaete chrysosporium on the Functional Diversity of Bacterial Communities Associated with Decaying Wood
Source: PLoS One. 2016 Jan 29;11(1):e0147100. doi: 10.1371/journal.pone.0147100 (PMC4732817; doi:10.1371/journal.pone.0147100)

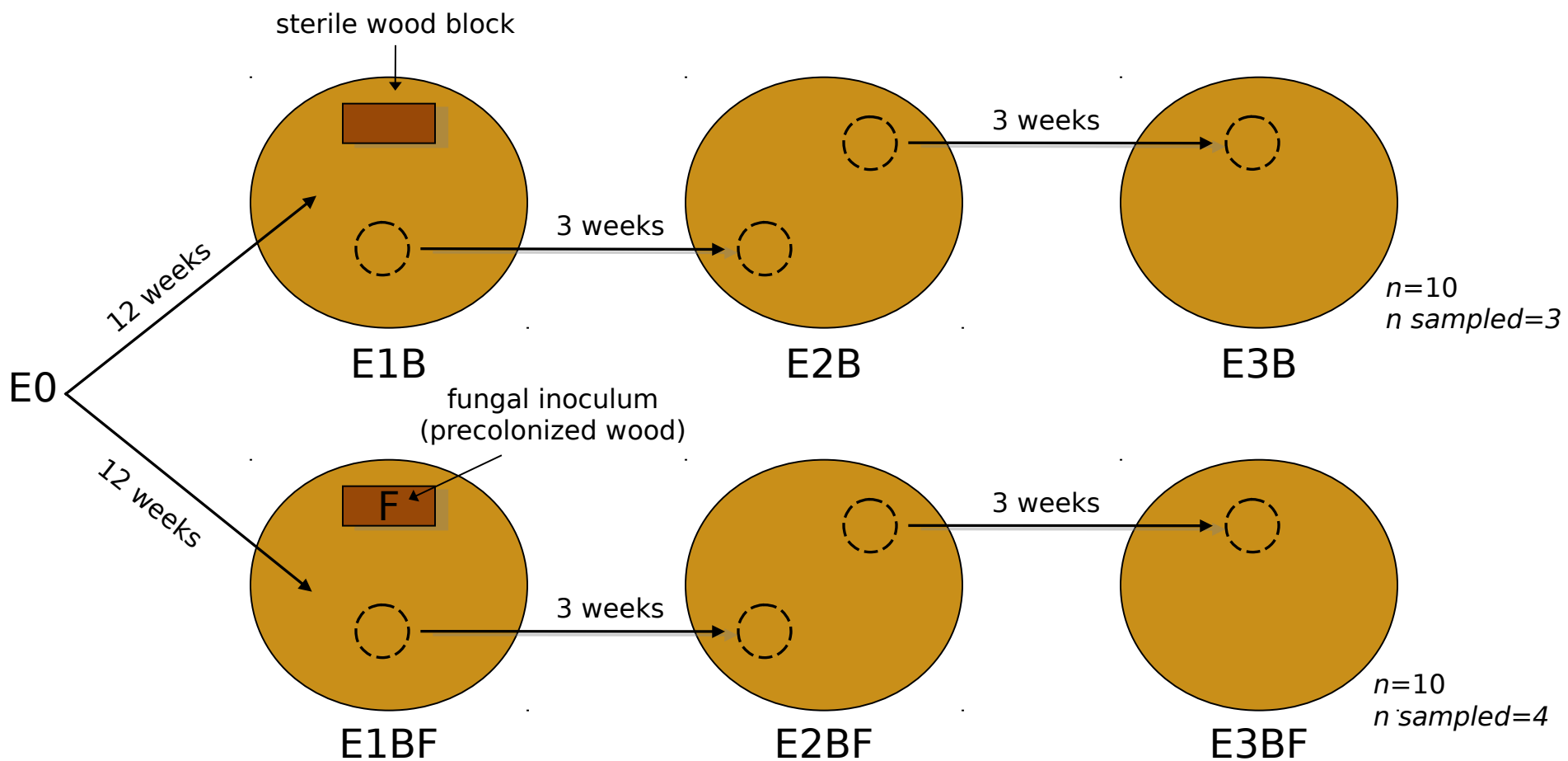

Supplement: S1 Fig — The diagram was adapted from [25]. E0, a microbial suspension extracted from forest soil, was used as the initial inoculum. E0 was mixed with sterile sawdust as growth matrix, in two conditions: including (BF) or not (B) the white-rot fungus P. chrysosporium previously inoculated on a beech wood block (F). After twelve weeks of incubation (E1), an enrichment was performed every three weeks (from E1 to E3), using a fraction of colonized sawdust sampled from a microcosm to inoculate a new sterile one. (PDF) [file pone.0147100.s001.pdf]

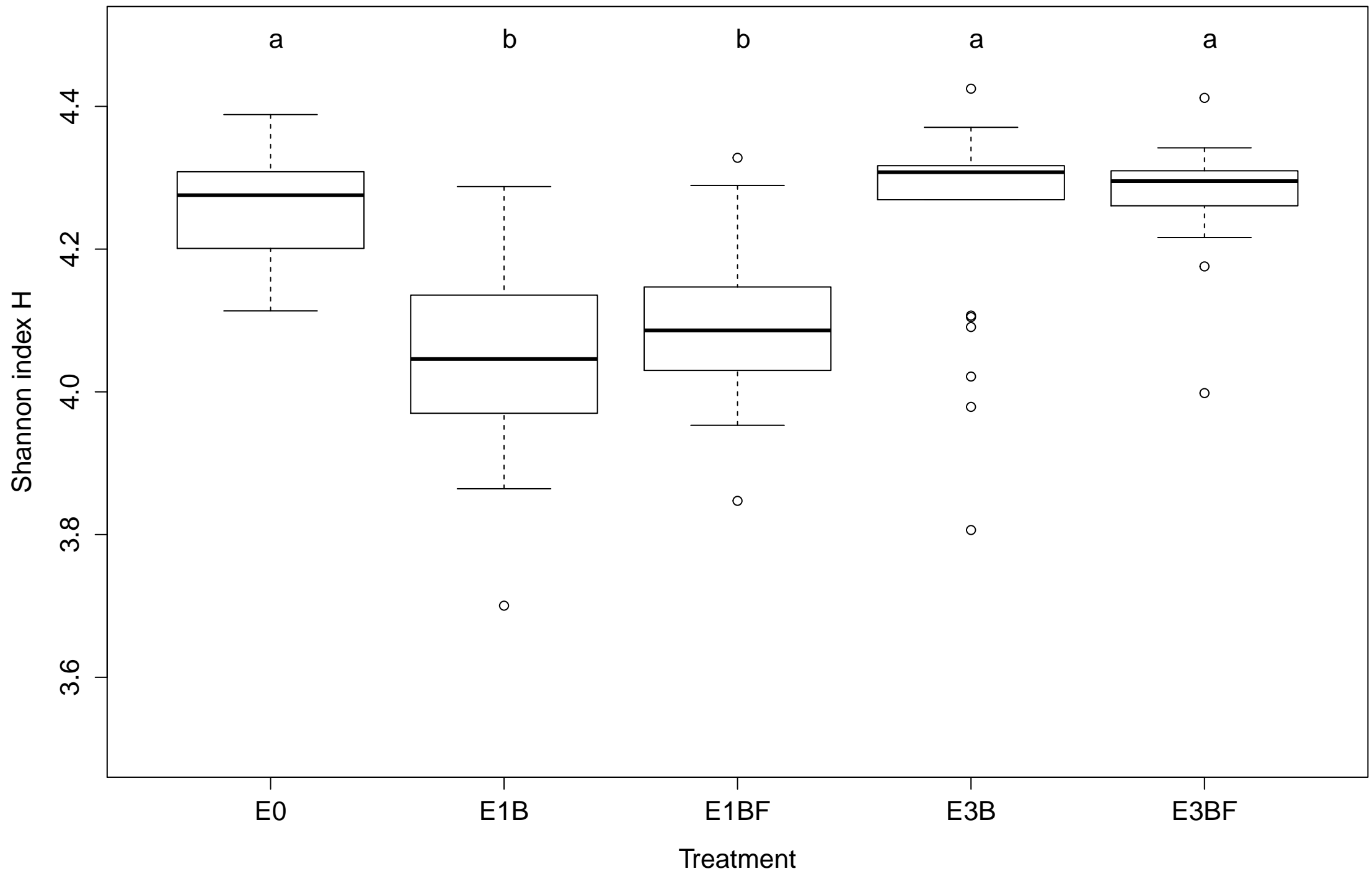

Supplement: S2 Fig — Different letters above boxplots indicate significant difference based on non-parametric Kruskal-Wallis test (p<0.05). (PDF) [file pone.0147100.s002.pdf]
