# Supplementary material for: Impact of Phanerochaete chrysosporium on the Functional Diversity of Bacterial Communities Associated with Decaying Wood
Source: PLoS One. 2016 Jan 29;11(1):e0147100. doi: 10.1371/journal.pone.0147100 (PMC4732817; doi:10.1371/journal.pone.0147100)
Supplement: S1 Table — The assignment was performed using the EzTaxon-e server [35] and on the basis of 16S rRNA gene sequences (>800 nucleotides). The 125 strains randomly selected for functional characterization are highlighted in gray. (PDF) [file pone.0147100.s003.pdf]

Table_S1

treatment	strains	closely related species	similarity score (%)	accession number
E0	E0_1	*Dyella koreensis*	98.95	KM604797
E0	E0_2	*Burkholderia sediminicola*	99.65	KM604798
E0	E0_3	*Collimonas pratensis*	99.66	KM604799
E0	E0_4	*Burkholderia sediminicola*	99.65	KM604800
E0	E0_5	*Burkholderia bryophila*	97.89	KM604801
E0	E0_6	*Burkholderia sordidicola*	98.11	KM604802
E0	E0_7	*Burkholderia bryophila*	97.88	KM604803
E0	E0_8	*Burkholderia caledonica*	98.59	KM604804
E0	E0_9	*Collimonas pratensis*	98.95	KM604805
E0	E0_10	*Dyella koreensis*	98.61	KM604806
E0	E0_11	*Dyella koreensis*	98.49	KM604807
E0	E0_12	*Burkholderia sediminicola*	99.65	KM604808
E0	E0_13	*Burkholderia bryophila*	97.90	KM604809
E0	E0_14	*Burkholderia phenazinium*	99.41	KM604810
E0	E0_15	*Burkholderia sordidicola*	98.95	KM604811
E0	E0_20	*Burkholderia bryophila*	99.30	KM604812
E0	E0_21	*Burkholderia phenazinium*	99.41	KM604813
E0	E0_22	*Burkholderia sediminicola*	99.65	KM604814
E0	E0_23	*Collimonas pratensis*	99.77	KM604815
E0	E0_24	*Collimonas pratensis*	99.53	KM604816
E0	E0_25	*Burkholderia sediminicola*	99.65	KM604817
E0	E0_27	*Burkholderia phenazinium*	98.81	KM604818
E0	E0_28	*Burkholderia phenazinium*	99.29	KM604819
E0	E0_29	*Burkholderia sordidicola*	97.21	KM604820
E0	E0_30	*Burkholderia sediminicola*	99.30	KM604821
E0	E0_31	*Burkholderia sediminicola*	99.65	KM604822
E0	E0_32	*Dyella koreensis*	98.49	KM604823
E0	E0_33	*Dyella koreensis*	98.50	KM604824
E0	E0_34	*Burkholderia phenazinium*	99.41	KM604825
E0	E0_35	*Dyella koreensis*	98.49	KM604826
E0	E0_36	*Burkholderia bryophila*	97.67	KM604827
E0	E0_37	*Burkholderia sordidicola*	97.79	KM604828
E0	E0_38	*Burkholderia bryophila*	98.84	KM604829
E0	E0_39	*Dyella kyungheensis*	98.37	KM604830
E0	E0_40	*Burkholderia sediminicola*	99.65	KM604831
E0	E0_41	*Burkholderia sediminicola*	99.53	KM604832
E0	E0_42	*Collimonas fungivorans*	98.82	KM604833
E0	E0_43	*Burkholderia bryophila*	99.06	KM604834
E0	E0_44	*Burkholderia sediminicola*	99.65	KM604835
E0	E0_45	*Burkholderia sediminicola*	99.65	KM604836
E0	E0_46	*Burkholderia phenazinium*	99.64	KM604837
E0	E0_47	*Burkholderia phenazinium*	99.41	KM604838
E1B	E1B3_1	*Luteibacter rhizovicinus*	99.77	KM604839
E1B	E1B3_2	*Burkholderia diazotrophica*	97.71	KM604840
E1B	E1B3_3	*Burkholderia diazotrophica*	97.67	KM604841
E1B	E1B3_4	*Burkholderia diazotrophica*	97.43	KM604842
E1B	E1B3_5	*Luteibacter rhizovicinus*	99.30	KM604843
E1B	E1B3_6	*Burkholderia diazotrophica*	97.44	KM604844
E1B	E1B3_7	*Luteibacter rhizovicinus*	99.88	KM604845
E1B	E1B3_8	*Burkholderia diazotrophica*	97.21	KM604846
E1B	E1B3_10	*Burkholderia sordidicola*	98.73	KM604847
E1B	E1B3_11	*Burkholderia diazotrophica*	97.34	KM604848

E1B	E1B3_12	*Luteibacter rhizovicinus*	99.42	KM604849
E1B	E1B3_13	*Burkholderia diazotrophica*	97.55	KM604850
E1B	E1B3_14	*Burkholderia diazotrophica*	97.56	KM604851
E1B	E1B4_1	*Luteibacter rhizovicinus*	99.42	KM604852
E1B	E1B4_2	*Burkholderia sordidicola*	97.55	KM604853
E1B	E1B4_3	*Burkholderia sordidicola*	98.61	KM604854
E1B	E1B4_4	*Luteibacter rhizovicinus*	99.77	KM604855
E1B	E1B4_5	*Rhizobium alamii*	98.89	KM604856
E1B	E1B4_6	*Burkholderia bryophila*	99.41	KM604857
E1B	E1B4_7	*Burkholderia diazotrophica*	97.45	KM604858
E1B	E1B4_8	*Burkholderia sordidicola*	98.59	KM604859
E1B	E1B4_9	*Burkholderia sordidicola*	98.59	KM604860
E1B	E1B4_10	*Burkholderia sordidicola*	98.72	KM604861
E1B	E1B4_11	*Burkholderia sordidicola*	98.83	KM604862
E1B	E1B4_12	*Luteibacter rhizovicinus*	99.77	KM604863
E1B	E1B4_13	*Luteibacter rhizovicinus*	99.77	KM604864
E1B	E1B4_14	*Burkholderia sordidicola*	98.71	KM604865
E1B	E1B5_1	*Burkholderia diazotrophica*	97.67	KM604866
E1B	E1B5_2	*Burkholderia diazotrophica*	97.44	KM604867
E1B	E1B5_3	*Luteibacter rhizovicinus*	100.00	KM604868
E1B	E1B5_4	*Luteibacter rhizovicinus*	99.43	KM604869
E1B	E1B5_5	*Burkholderia diazotrophica*	97.45	KM604870
E1B	E1B5_6	*Luteibacter rhizovicinus*	99.54	KM604871
E1B	E1B5_7	*Luteibacter rhizovicinus*	99.65	KM604872
E1B	E1B5_8	*Luteibacter rhizovicinus*	99.31	KM604873
E1B	E1B5_9	*Luteibacter rhizovicinus*	99.77	KM604874
E1B	E1B5_11	*Burkholderia terrae*	95.47	KM604875
E1B	E1B5_12	*Luteibacter rhizovicinus*	99.77	KM604876
E1B	E1B5_13	*Luteibacter rhizovicinus*	99.31	KM604877
E1B	E1B5_14	*Luteibacter rhizovicinus*	99.31	KM604878
E1BF	E1BF3_1	*Micrococcus yunnanensis*	99.88	KM604879
E1BF	E1BF3_2	*Luteibacter rhizovicinus*	99.88	KM604880
E1BF	E1BF3_3	*Luteibacter rhizovicinus*	99.65	KM604881
E1BF	E1BF3_4	*Luteibacter rhizovicinus*	100.00	KM604882
E1BF	E1BF3_5	*Luteibacter rhizovicinus*	99.42	KM604883
E1BF	E1BF3_6	*Burkholderia diazotrophica*	95.77	KM604884
E1BF	E1BF3_7	*Burkholderia diazotrophica*	97.56	KM604885
E1BF	E1BF3_8	*Burkholderia diazotrophica*	97.69	KM604886
E1BF	E1BF3_9	*Burkholderia diazotrophica*	97.43	KM604887
E1BF	E1BF3_10	*Burkholderia diazotrophica*	97.32	KM604888
E1BF	E1BF3_11	*Burkholderia bryophila*	98.83	KM604889
E1BF	E1BF3_12	*Burkholderia bryophila*	98.83	KM604890
E1BF	E1BF6_1	*Burkholderia bryophila*	99.06	KM604891
E1BF	E1BF6_2	*Burkholderia bryophila*	99.06	KM604892
E1BF	E1BF6_3	*Burkholderia diazotrophica*	97.80	KM604893
E1BF	E1BF6_4	*Luteibacter rhizovicinus*	99.30	KM604894
E1BF	E1BF6_5	*Burkholderia sediminicola*	99.65	KM604895
E1BF	E1BF6_6	*Burkholderia diazotrophica*	97.43	KM604896
E1BF	E1BF6_7	*Burkholderia bryophila*	98.70	KM604897
E1BF	E1BF6_8	*Dyella koreensis*	98.60	KM604898
E1BF	E1BF6_9	*Burkholderia diazotrophica*	97.43	KM604899
E1BF	E1BF6_10	*Burkholderia diazotrophica*	95.64	KM604900
E1BF	E1BF6_11	*Burkholderia bryophila*	99.53	KM604901

E1BF	E1BF6_12	*Luteibacter rhizovicinus*	99.42	KM604902
E1BF	E1BF7_1	*Burkholderia diazotrophica*	95.72	KM604903
E1BF	E1BF7_2	*Luteibacter rhizovicinus*	100.00	KM604904
E1BF	E1BF7_3	*Luteibacter rhizovicinus*	99.54	KM604905
E1BF	E1BF7_4	*Luteibacter rhizovicinus*	99.42	KM604906
E1BF	E1BF7_5	*Luteibacter rhizovicinus*	99.42	KM604907
E1BF	E1BF7_6	*Burkholderia diazotrophica*	97.56	KM604908
E1BF	E1BF7_7	*Burkholderia diazotrophica*	95.84	KM604909
E1BF	E1BF7_8	*Burkholderia diazotrophica*	95.98	KM604910
E1BF	E1BF7_9	*Luteibacter rhizovicinus*	99.88	KM604911
E1BF	E1BF7_10	*Burkholderia bryophila*	99.41	KM604912
E1BF	E1BF7_11	*Burkholderia diazotrophica*	95.72	KM604913
E1BF	E1BF7_12	*Luteibacter rhizovicinus*	99.77	KM604914
E1BF	E1BF9_1	*Burkholderia diazotrophica*	97.56	KM604915
E1BF	E1BF9_2	*Burkholderia diazotrophica*	97.43	KM604916
E1BF	E1BF9_3	*Luteibacter rhizovicinus*	99.88	KM604917
E1BF	E1BF9_4	*Luteibacter rhizovicinus*	99.08	KM604918
E1BF	E1BF9_5	*Burkholderia diazotrophica*	97.32	KM604919
E1BF	E1BF9_6	*Burkholderia diazotrophica*	97.44	KM604920
E1BF	E1BF9_7	*Burkholderia diazotrophica*	97.56	KM604921
E1BF	E1BF9_8	*Burkholderia diazotrophica*	97.56	KM604922
E1BF	E1BF9_9	*Burkholderia diazotrophica*	97.08	KM604923
E1BF	E1BF9_10	*Burkholderia bryophila*	98.84	KM604924
E1BF	E1BF9_11	*Luteibacter rhizovicinus*	99.65	KM604925
E1BF	E1BF9_12	*Luteibacter rhizovicinus*	99.77	KM604926
E2B	E2B3_1	*Burkholderia diazotrophica*	97.33	KM604927
E2B	E2B3_2	*Luteibacter rhizovicinus*	99.42	KM604928
E2B	E2B3_3	*Luteibacter rhizovicinus*	99.54	KM604929
E2B	E2B3_4	*Luteibacter rhizovicinus*	99.88	KM604930
E2B	E2B3_5	*Luteibacter rhizovicinus*	99.77	KM604931
E2B	E2B3_6	*Luteibacter rhizovicinus*	99.42	KM604932
E2B	E2B3_7	*Luteibacter rhizovicinus*	99.88	KM604933
E2B	E2B3_8	*Luteibacter rhizovicinus*	99.77	KM604934
E2B	E2B3_9	*Luteibacter rhizovicinus*	99.88	KM604935
E2B	E2B3_10	*Luteibacter rhizovicinus*	99.89	KM604936
E2B	E2B3_11	*Luteibacter rhizovicinus*	99.77	KM604937
E2B	E2B3_12	*Luteibacter rhizovicinus*	99.77	KM604938
E2B	E2B3_13	*Luteibacter rhizovicinus*	99.77	KM604939
E2B	E2B3_14	*Luteibacter rhizovicinus*	99.66	KM604940
E2B	E2B4_1	*Luteibacter rhizovicinus*	99.77	KM604941
E2B	E2B4_2	*Burkholderia sordidicola*	97.80	KM604942
E2B	E2B4_3	*Luteibacter rhizovicinus*	99.77	KM604943
E2B	E2B4_4	*Burkholderia diazotrophica*	95.52	KM604944
E2B	E2B4_5	*Burkholderia bryophila*	99.30	KM604945
E2B	E2B4_6	*Luteibacter rhizovicinus*	99.54	KM604946
E2B	E2B4_7	*Luteibacter rhizovicinus*	99.43	KM604947
E2B	E2B4_8	*Luteibacter rhizovicinus*	99.66	KM604948
E2B	E2B4_9	*Burkholderia caledonica*	97.19	KM604949
E2B	E2B4_10	*Burkholderia bryophila*	99.30	KM604950
E2B	E2B4_11	*Luteibacter rhizovicinus*	99.54	KM604951
E2B	E2B4_12	*Luteibacter rhizovicinus*	99.65	KM604952
E2B	E2B4_13	*Burkholderia bryophila*	99.19	KM604953
E2B	E2B4_14	*Burkholderia bryophila*	99.19	KM604954

E2B	E2B5_1	*Burkholderia bryophila*	99.08	KM604955
E2B	E2B5_2	*Luteibacter rhizovicinus*	99.88	KM604956
E2B	E2B5_3	*Luteibacter rhizovicinus*	99.42	KM604957
E2B	E2B5_4	*Luteibacter rhizovicinus*	99.77	KM604958
E2B	E2B5_5	*Variovorax boronicumulans*	99.30	KM604959
E2B	E2B5_6	*Achromobacter spanius*	99.76	KM604960
E2B	E2B5_7	*Achromobacter spanius*	99.65	KM604961
E2B	E2B5_8	*Achromobacter spanius*	99.65	KM604962
E2B	E2B5_9	*Achromobacter spanius*	99.53	KM604963
E2B	E2B5_10	*Achromobacter spanius*	99.53	KM604964
E2B	E2B5_11	*Achromobacter spanius*	99.65	KM604965
E2B	E2B5_12	*Achromobacter spanius*	99.53	KM604966
E2B	E2B5_13	*Variovorax boronicumulans*	99.18	KM604967
E2B	E2B5_14	*Achromobacter spanius*	99.65	KM604968
E2BF	E2BF3_1	*Burkholderia bryophila*	98.95	KM604969
E2BF	E2BF3_2	*Burkholderia bryophila*	99.41	KM604970
E2BF	E2BF3_3	*Burkholderia bryophila*	98.94	KM604971
E2BF	E2BF3_4	*Luteibacter rhizovicinus*	99.65	KM604972
E2BF	E2BF3_5	*Burkholderia diazotrophica*	96.24	KM604973
E2BF	E2BF3_6	*Burkholderia bryophila*	98.71	KM604974
E2BF	E2BF3_7	*Luteibacter rhizovicinus*	99.42	KM604975
E2BF	E2BF3_8	*Luteibacter rhizovicinus*	99.88	KM604976
E2BF	E2BF3_9	*Burkholderia bryophila*	98.71	KM604977
E2BF	E2BF3_10J	*Luteibacter rhizovicinus*	99.88	KM604978
E2BF	E2BF3_10B	*Rhizobium jaguaris*	99.25	KM604979
E2BF	E2BF3_11	*Burkholderia bryophila*	98.94	KM604980
E2BF	E2BF3_12	*Burkholderia fungorum*	98.47	KM604981
E2BF	E2BF6_1	*Luteibacter rhizovicinus*	99.65	KM604982
E2BF	E2BF6_2	*Achromobacter spanius*	99.53	KM604983
E2BF	E2BF6_3	*Dyella koreensis*	98.83	KM604984
E2BF	E2BF6_4	*Burkholderia terrae*	95.52	KM604985
E2BF	E2BF6_5	*Burkholderia bryophila*	98.59	KM604986
E2BF	E2BF6_6	*Luteibacter rhizovicinus*	99.65	KM604987
E2BF	E2BF6_7	*Dyella kyungheensis*	98.49	KM604988
E2BF	E2BF6_8	*Burkholderia sordidicola*	97.89	KM604989
E2BF	E2BF6_9	*Burkholderia bryophila*	98.71	KM604990
E2BF	E2BF6_10	*Dyella kyungheensis*	98.37	KM604991
E2BF	E2BF6_11	*Luteibacter rhizovicinus*	100.00	KM604992
E2BF	E2BF6_12	*Luteibacter rhizovicinus*	99.65	KM604993
E2BF	E2BF7_1	*Burkholderia bryophila*	98.82	KM604994
E2BF	E2BF7_2	*Luteibacter rhizovicinus*	99.77	KM604995
E2BF	E2BF7_3	*Luteibacter rhizovicinus*	99.54	KM604996
E2BF	E2BF7_4	*Burkholderia bryophila*	98.94	KM604997
E2BF	E2BF7_5	*Luteibacter rhizovicinus*	100.00	KM604998
E2BF	E2BF7_6	*Luteibacter rhizovicinus*	100.00	KM604999
E2BF	E2BF7_7	*Luteibacter rhizovicinus*	100.00	KM605000
E2BF	E2BF7_8	*Luteibacter rhizovicinus*	99.88	KM605001
E2BF	E2BF7_9	*Luteibacter rhizovicinus*	100.00	KM605002
E2BF	E2BF7_10	*Rhizobium alamii*	98.88	KM605003
E2BF	E2BF7_11	*Luteibacter rhizovicinus*	99.65	KM605004
E2BF	E2BF7_12	*Luteibacter rhizovicinus*	99.42	KM605005
E2BF	E2BF9_1	*Burkholderia bryophila*	99.29	KM605006
E2BF	E2BF9_2	*Luteibacter rhizovicinus*	100.00	KM605007

E2BF	E2BF9_3	*Luteibacter rhizovicinus*	99.65	KM605008
E2BF	E2BF9_4	*Burkholderia bryophila*	99.18	KM605009
E2BF	E2BF9_5	*Burkholderia bryophila*	99.41	KM605010
E2BF	E2BF9_6	*Luteibacter rhizovicinus*	99.77	KM605011
E2BF	E2BF9_7	*Dyella koreensis*	98.84	KM605012
E2BF	E2BF9_8	*Burkholderia bryophila*	99.41	KM605013
E2BF	E2BF9_9	*Luteibacter rhizovicinus*	99.53	KM605014
E2BF	E2BF9_10	*Burkholderia sartisoli*	97.19	KM605015
E2BF	E2BF9_11	*Burkholderia bryophila*	98.94	KM605016
E2BF	E2BF9_12	*Burkholderia bryophila*	99.29	KM605017
E3B	E3B3_1	*Burkholderia sediminicola*	99.53	KM605018
E3B	E3B3_2	*Burkholderia bryophila*	99.29	KM605019
E3B	E3B3_3	*Burkholderia sediminicola*	99.77	KM605020
E3B	E3B3_4	*Burkholderia sediminicola*	99.65	KM605021
E3B	E3B3_5	*Luteibacter rhizovicinus*	99.77	KM605022
E3B	E3B3_6	*Luteibacter rhizovicinus*	99.42	KM605023
E3B	E3B3_7	*Burkholderia caledonica*	97.07	KM605024
E3B	E3B3_8	*Luteibacter rhizovicinus*	99.77	KM605025
E3B	E3B3_9	*Burkholderia sediminicola*	99.65	KM605026
E3B	E3B3_10	*Luteibacter rhizovicinus*	99.88	KM605027
E3B	E3B3_11	*Burkholderia sediminicola*	99.65	KM605028
E3B	E3B3_12	*Burkholderia bryophila*	99.53	KM605029
E3B	E3B3_13	*Burkholderia bryophila*	99.53	KM605030
E3B	E3B3_14	*Burkholderia bryophila*	99.29	KM605031
E3B	E3B4_1	*Dyella japonica*	99.77	KM605032
E3B	E3B4_2	*Burkholderia bryophila*	99.53	KM605033
E3B	E3B4_3	*Burkholderia bryophila*	99.42	KM605034
E3B	E3B4_4	*Luteibacter rhizovicinus*	99.77	KM605035
E3B	E3B4_5	*Burkholderia caledonica*	97.12	KM605036
E3B	E3B4_6	*Burkholderia caledonica*	96.98	KM605037
E3B	E3B4_7	*Burkholderia bryophila*	99.30	KM605038
E3B	E3B4_8	*Burkholderia caledonica*	97.20	KM605039
E3B	E3B4_9	*Burkholderia caledonica*	97.09	KM605040
E3B	E3B4_10	*Burkholderia caledonica*	97.19	KM605041
E3B	E3B4_11	*Burkholderia caledonica*	97.09	KM605042
E3B	E3B4_12	*Burkholderia caledonica*	97.19	KM605043
E3B	E3B4_13	*Burkholderia caledonica*	97.10	KM605044
E3B	E3B4_14	*Burkholderia caledonica*	96.86	KM605045
E3B	E3B5_1	*Burkholderia caledonica*	97.07	KM605046
E3B	E3B5_2	*Burkholderia caledonica*	97.19	KM605047
E3B	E3B5_3	*Burkholderia sartisoli*	97.19	KM605048
E3B	E3B5_4	*Burkholderia caledonica*	96.84	KM605049
E3B	E3B5_5	*Burkholderia caledonica*	97.10	KM605050
E3B	E3B5_6	*Burkholderia caledonica*	97.10	KM605051
E3B	E3B5_7	*Cupriavidus gilardii*	97.32	KM605052
E3B	E3B5_8	*Burkholderia sartisoli*	97.19	KM605053
E3B	E3B5_9	*Burkholderia caledonica*	97.19	KM605054
E3B	E3B5_10	*Burkholderia caledonica*	97.07	KM605055
E3B	E3B5_11	*Burkholderia sartisoli*	97.19	KM605056
E3B	E3B5_12	*Burkholderia sartisoli*	98.04	KM605057
E3B	E3B5_13	*Burkholderia caledonica*	97.18	KM605058
E3B	E3B5_14	*Burkholderia caledonica*	97.19	KM605059
E3BF	E3BF3_1	*Burkholderia fungorum*	98.25	KM605060

E3BF	E3BF3_2	*Burkholderia bryophila*	99.41	KM605061
E3BF	E3BF3_3	*Burkholderia bryophila*	98.71	KM605062
E3BF	E3BF3_4	*Burkholderia caledonica*	97.19	KM605063
E3BF	E3BF3_5	*Burkholderia caledonica*	96.95	KM605064
E3BF	E3BF3_6	*Burkholderia bryophila*	98.82	KM605065
E3BF	E3BF3_7	*Burkholderia bryophila*	99.53	KM605066
E3BF	E3BF3_8	*Dyella koreensis*	98.85	KM605067
E3BF	E3BF3_9	*Burkholderia bryophila*	98.83	KM605068
E3BF	E3BF3_10	*Burkholderia bryophila*	98.94	KM605069
E3BF	E3BF3_11	*Burkholderia sartisoli*	97.19	KM605070
E3BF	E3BF3_12	*Burkholderia sartisoli*	97.19	KM605071
E3BF	E3BF6_1	*Burkholderia bryophila*	98.59	KM605072
E3BF	E3BF6_2	*Burkholderia caledonica*	96.95	KM605073
E3BF	E3BF6_3	*Burkholderia bryophila*	98.94	KM605074
E3BF	E3BF6_4	*Dyella kyungheensis*	98.25	KM605075
E3BF	E3BF6_5	*Burkholderia sediminicola*	99.53	KM605076
E3BF	E3BF6_6	*Burkholderia caledonica*	96.62	KM605077
E3BF	E3BF6_7	*Burkholderia bryophila*	98.82	KM605078
E3BF	E3BF6_8	*Burkholderia bryophila*	98.94	KM605079
E3BF	E3BF6_9	*Dyella koreensis*	98.60	KM605080
E3BF	E3BF6_10	*Burkholderia bryophila*	98.94	KM605081
E3BF	E3BF6_11	*Burkholderia bryophila*	98.82	KM605082
E3BF	E3BF6_12	*Burkholderia bryophila*	99.41	KM605083
E3BF	E3BF7_1	*Dyella japonica*	99.07	KM605084
E3BF	E3BF7_2	*Burkholderia bryophila*	98.57	KM605085
E3BF	E3BF7_3	*Burkholderia bryophila*	99.53	KM605086
E3BF	E3BF7_4	*Burkholderia sediminicola*	99.53	KM605087
E3BF	E3BF7_5	*Burkholderia bryophila*	98.35	KM605088
E3BF	E3BF7_6	*Burkholderia bryophila*	98.59	KM605089
E3BF	E3BF7_7	*Burkholderia ginsengisoli*	99.30	KM605090
E3BF	E3BF7_8	*Burkholderia bryophila*	98.37	KM605091
E3BF	E3BF7_9	*Burkholderia bryophila*	98.25	KM605092
E3BF	E3BF7_10	*Burkholderia bryophila*	98.47	KM605093
E3BF	E3BF7_11	*Burkholderia phenazinium*	99.53	KM605094
E3BF	E3BF7_12	*Burkholderia ginsengisoli*	99.18	KM605095
E3BF	E3BF9_1	*Burkholderia caledonica*	97.29	KM605096
E3BF	E3BF9_2	*Dyella japonica*	99.65	KM605097
E3BF	E3BF9_3	*Burkholderia sediminicola*	99.53	KM605098
E3BF	E3BF9_4	*Burkholderia caledonica*	97.18	KM605099
E3BF	E3BF9_5	*Burkholderia sartisoli*	97.19	KM605100
E3BF	E3BF9_6	*Burkholderia sediminicola*	99.53	KM605101
E3BF	E3BF9_7	*Dyella japonica*	99.65	KM605102
E3BF	E3BF9_8	*Burkholderia caledonica*	97.06	KM605103
E3BF	E3BF9_9	*Burkholderia sartisoli*	97.19	KM605104
E3BF	E3BF9_10	*Burkholderia sordidicola*	98.12	KM605105
E3BF	E3BF9_11	*Burkholderia caledonica*	97.29	KM605106
E3BF	E3BF9_12	*Dyella japonica*	99.77	KM605107
