# Supplementary material for: Impact of Phanerochaete chrysosporium on the Functional Diversity of Bacterial Communities Associated with Decaying Wood
Source: PLoS One. 2016 Jan 29;11(1):e0147100. doi: 10.1371/journal.pone.0147100 (PMC4732817; doi:10.1371/journal.pone.0147100)
Supplement: S2 Table — The comparison was based on 16S rRNA gene sequences and using the RDP Library Compare tool version 2.6 with 95% confidence threshold, except for Blomqvist et al. [50] in which no sequences were available but only taxonomic identification. (PDF) [file pone.0147100.s004.pdf]

	**present study**	**Folman et al. 2008**	**Blomqvist et al. 2014**	**Zhang et al. 2008**	**Valaskova et al. 2009**	**Valaskova et al. 2009**
	**Culture-dependent**	**Culture-dependent**	**Culture-dependent**	**Culture-independent**	**Culture-dependent**	**Culture-independent**
Proteobacteria phylum	99.63	82.61	98.55	51.85	75.00	55.22
Burkholderiaceae family	59.11	34.78	8.70	0.00	27.08	13.43
Xanthomonadaceae family	35.32	4.35	52.17	1.23	22.92	3.73
Alcaligenaceae family	3.35	0.87	0.00	0.00	0.00	0.00
Comamonadaceae family	0.74	0.87	0.00	1.23	0.00	0.00
Rhizobiaceae family	1.12	0.00	0.00	0.00	0.00	0.00
Micrococcaceae family	0.37	0.00	0.00	0.00	0.00	0.00
